# Cell cycle commitment in budding yeast emerges from the cooperation of multiple bistable switches

**DOI:** 10.1098/rsob.110009

**Published:** 2011-11

**Authors:** Tongli Zhang, Bernhard Schmierer, Béla Novák

**Affiliations:** Oxford Centre for Integrative Systems Biology (OCISB), Department of Biochemistry, University of Oxford, South Parks Road, Oxford OX1 3QU, UK

**Keywords:** bistability, budding yeast, decision making, dynamical systems, pheromone response, START transition

## Abstract

The start-transition (START) in the G1 phase marks the point in the cell cycle at which a yeast cell initiates a new round of cell division. Once made, this decision is irreversible and the cell is committed to progressing through the entire cell cycle, irrespective of arrest signals such as pheromone. How commitment emerges from the underlying molecular interaction network is poorly understood. Here, we perform a dynamical systems analysis of an established cell cycle model, which has never been analysed from a commitment perspective. We show that the irreversibility of the START transition and subsequent commitment can be consistently explained in terms of the interplay of multiple bistable molecular switches. By applying an existing mathematical model to a novel problem and by expanding the model in a self-consistent manner, we achieve several goals: we bring together a large number of experimental findings into a coherent theoretical framework; we increase the scope and the applicability of the original model; we give a systems level explanation of how the START transition and the cell cycle commitment arise from the dynamical features of the underlying molecular interaction network; and we make clear, experimentally testable predictions.

## 2. Introduction

Yeast cells enter the cell division cycle depending on cell mass as well as on environmental cues such as nutrient availability, and they arrest cycling in response to factors such as mating pheromone. Signal transduction cascades interact with and affect the cell cycle control network to either promote or halt entry into a new round of cell division [1]. The decision to initiate a new cycle is made at the start-transition (START) in the G1 phase [2]. START is a point of no return: post-START cells are irrevocably committed to cell division and will finish the cycle, irrespective of environmental cues. This characteristic has led to the initial conceptual definition of START as the point in the cell cycle after which a yeast cell no longer responds with arrest to mating pheromone [3]. Such cells are said to be committed and, despite the presence of pheromone, progress normally through late G1, S, G2 and M to finally arrest early in G1 of the following cycle, indicating that, at this point, pheromone sensitivity is restored. Robust decision-making is required to guarantee that the two incompatible cell fates—pheromone-induced mating and cell division—are never realized simultaneously.

At the molecular level, pheromone binds to the receptor Ste2 to initiate a mitogen-activated protein kinase (MAPK) cascade. The terminal kinase in this cascade, Fus3, activates the transcription factor Ste12, which in turn induces the expression of mating genes. One of these targets, the Cdk1-inhibitor Far1 [4], pheromone-dependently interacts with Cln1/2/3:Cdk1 complexes [5] to inhibit their activity [6], but not to B-type cyclin:Cdk1 complexes. Far1 is crucially involved in arresting cells in response to pheromone [7] and requires Fus3-dependent phosphorylation for full activity [8,9]. Transcriptional induction and activation of Far1 by Ste12 depend on the assembly of the pheromone-induced MAPK cascade on the scaffolding protein Ste5, which is recruited to the membrane in response to pheromone. Membrane recruitment activates Ste5 [10], whereas phosphorylation by Cln1/2:Cdk1 complexes inactivates the protein, thus blocking Far1 activation, and also transcriptional activation of other Ste12 targets [11]. In addition, direct phosphorylation of Far1 by Cln1/2:Cdk1 promotes Far1 degradation [12]. In summary, Cln1/2:Cdk1 complexes inhibit their own inhibitor Far1 in a dual manner—by blocking its synthesis and activation, and by promoting its degradation.

While the topologies of the molecular interaction networks of both the cell cycle engine and of pheromone signalling are becoming increasingly well-defined, the fundamental questions of how the molecular interaction network gives rise to robust decision-making and to commitment have only recently begun to be addressed [13,14]. From a dynamical systems point of view, the irreversible START transition can be explained by the presence of a positive feedback loop in the underlying biochemical control network [13], which can cause switch-like behaviour [15,16]. However, additional negative feedback loops counteract these self-enhancing processes [17,18]. How cells balance these contradictory forces remains poorly understood.

Here, we address these issues by analysing and expanding the model of Chen *et al.* [19] (hereafter ‘Chen's Model’), the most comprehensive mathematical model of the yeast cell cycle to date. This model combines an ambitious scope with extremely careful validation against a large number of datasets from a large number of cell cycle mutants, and is thus well constrained. By integrating a large proportion of the available knowledge on the interactions in the budding yeast cell cycle, its original parametrization successfully captures the behaviour of more than 130 mutant strains. Chen's model has never been analysed from a commitment perspective, and we first use the model in its original form and parametrization to simulate and corroborate recent experimental findings. We showcase that a published model can be successfully applied to novel problems, and can be expanded in its scope in a self-consistent manner. In doing so, we hope to encourage a wider use of *existing* mathematical models both by experimentalists (to predict the outcomes of experiments) and by modellers (to use newly available datasets to challenge and improve published models).

Our simulations explain the notion that Cln2 auto-activation is important for a robust START transition [13] at the level of the comprehensive cell cycle control system. We demonstrate that cell cycle commitment is a systems property and propose that commitment emerges from the cooperation of several bistable switches in the underlying, feedback-controlled reaction network. We go on to demonstrate that commitment is maintained by two fundamentally distinct mechanisms, depending on the post-START cell cycle stage.

Two mutations in components of the pheromone pathway (Far1-S87P and Ste5-8A) have been described, which both render the respective protein resistant to the inhibitory effect of Cln1/2:Cdk1-dependent phosphorylation; however, they differ in their phenotypes. Only our theoretical results are not only consistent with the observed differential behaviour of these mutants, but in addition make a testable prediction as to how the abnormal cell cycle arrest, which is observed in a fraction of Ste5-8A cells, could arise.

## 3. Results

**Figure 1.**
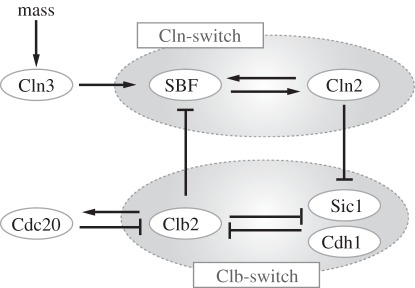
Some important feedback loops in the budding yeast cell cycle control network. Cln3, Cln2 and Clb2 denote the corresponding Cyclin:Cdk1 complexes. Briefly, Cln3 initiates the cell cycle in a cell-mass-dependent fashion by activating the transcription factor SBF, which triggers the expression of Cln2. Cln2 activates its own transcription (Cln-switch). By inhibiting both Sic1 and Cdh1, Cln2 promotes the activity of Clb2, which self-activates by further inhibiting Sic1 and Cdh1 (Clb-switch). Once active, Clb2 represses SBF and blocks Cln2 expression. At late cell cycle stages, Clb2 induces Cdc20, which triggers Clb2 degradation. A comprehensive description of the cell cycle control network used for modelling is given by Chen *et al*. [19].

For simplicity, ‘Cln2’ and ‘Clb2’ are used throughout as shorthand for Cln1/2:Cdk1 complexes and Clb1/2:Cdk1 complexes, respectively. Chen's model explains cell cycle dynamics in budding yeast in terms of interconnected positive and double-negative feedback loops, which can act as bistable switches (figure 1). Cln2 activity is controlled by a positive feedback loop: Cln2 phosphorylates and induces the nuclear export of Whi5 to activate the transcription factor SBF [20,21], which in turn promotes the expression of *CLN2* and also of *CLN1* [22,23]. We refer to this positive feedback loop as the ‘Cln-switch’. By contrast, Clb2 is controlled by double-negative feedback loops: Clb2 inactivates its stoichiometric inhibitor Sic1 [24] as well as the anaphase-promoting complex (APC) component Cdh1, which targets the cyclin-component of Clb2 (and also of Clb1) for degradation. We refer to these double-negative feedback loops as the ‘Clb-switch’. These network motifs interact with each other, the Cln-switch activating the Clb-switch and the Clb-switch inhibiting the Cln-switch in a negative feedback loop [17]. For a detailed description of the considerably more complex full model network, the model assumptions, the parameter values, etc., refer to the original publication of Chen's model [19] or the model webpage (the URL is given in §5.4).

### 3.1. Chen's model captures novel experimental results

Our first goal was to test whether Chen's model in its original form and parametrization could explain novel experimental findings that were unknown at the time of model conception. In a series of elegant experiments, Charvin *et al*. [13] (hereafter ‘Charvin's experiments’) have recently provided good evidence for a bistable Cln-switch, which had long been postulated on theoretical grounds [25], but not been demonstrated experimentally. Briefly, Cln1 and Cln2 were isolated from other regulatory interactions in the cell cycle machinery using a strain with the genetic background *cln3*Δ* bck2*Δ* GAL1-SIC1-4A MET3-CLN2*. This strain lacks both Cln3 and Bck2, contains a galactose-inducible version of the B-cyclin:Cdk1 inhibitor Sic1 (Sic1-4A), which is non-phosphorylatable and thus constitutively active, and a methionine-repressible *CLN2*. In this strain, the Clb2 switch is kept in the OFF state by *GAL-SIC1-4A*. To determine whether transiently expressed Cln2 would be sufficient to turn the Cln-switch ON, cells are initially arrested in early G1 in the presence of galactose and methionine (no Clb2 activity and no exogenous Cln2 expressed). Transient methionine removal causes a short pulse of expression of exogenous Cln2, which activates SBF. SBF then induces the expression of endogenous *CLN1* and *CLN2*, which are able to sustain their own expression through SBF. The experiment demonstrates an irreversible transition brought about by a transient signal. (In wild-type cells, the activity of Cln2 would be turned off once B-type cyclins are activated, which in the experimental strain is prevented by Sic1-4A expression, effectively isolating Cln2 from Clb2.)

**Figure 2.**
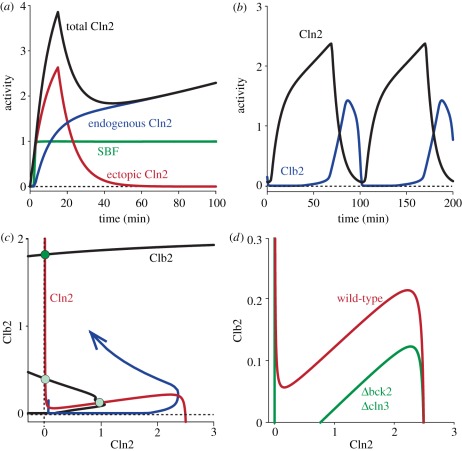
Dynamical systems analysis of START. (a) Time course simulation of Charvin's experiment. Fifteen minutes of expression of ectopic Cln2 in a *cln3*Δ* bck2*Δ* GAL1-SIC1-4A MET3-CLN2* background is sufficient to activate SBF, which induces self-sustained expression of endogenous Cln2; compare fig. 4*j* in [13]. Because Clb2 is kept inactive by Sic1-4A in this particular strain, Cln2 remains high. (*b*) Time course simulation of a wild-type cell. Cln2 expression precedes Clb2 expression. This temporal order is a consequence of Cln2-activating Clb2, and Clb2-inhibiting Cln2. Completion of the cell cycle is achieved by downregulation of Clb2 at later stages of the cell cycle, which resets the system to its original state. (c) Bifurcation analysis of a wild-type cell. Two one-parameter bifurcation curves are plotted on the same diagram (pseudo-phaseplane). Both the Cln2 bifurcation curve (red, Cln-switch) and the Clb2 bifurcation curve (black, Clb-switch) are bistable. The Clb-switch engages at a threshold concentration of Cln2 and is self-sustaining. This transition is irreversible (black curve). Cln2 activity (red curve) is inhibited by Clb2 activity and is high only if Clb2 is low. The time trajectory of the system (blue curve) starts from G1 with low levels of both Cln2 and Clb2 activity; the system moves in an anti-clockwise direction. As the Cln-switch engages, the trajectory shoots towards the stable upper branch of the Cln2 curve. High Cln2 activity triggers firing of the Clb-switch, which feeds back negatively to repress Cln2 activity, and the trajectory moves to the upper left towards the system's only stable steady state (green circle). This steady state is not reached, however, because processes at later stages of the cell cycle, such as Cdc20 activation, inactivate Clb2, which would cause the trajectory to move down to eventually reach its origin (not shown). (*d*) Bifurcation analysis. In *cln3*Δ* bck2*Δ** cells (green curve), the system is bistable even at zero Clb2 activity. This is the situation in Charvin's experiment. In wild-type cells, by contrast (red curve, identical to the red curve in (*c*)), a finite Clb2 activity is required to allow bistability. If such activity is absent, the steady state corresponding to low Cln2 activity is lost. The Cln-switch is stuck in the ON state.

To test whether Chen's model captures this behaviour, we simulated the experiment (compare the model simulation in figure 2*a* with fig. 4*j* in [13], which shows the accumulation of a fluorescent reporter protein driven by the *CLN2* promoter in *cln3*Δ* bck2*Δ* GAL1-SIC1-4A MET3-CLN2*). The model simulation was carried out with Chen's standard parameter set, except for parameters that had to be zeroed to account for the specific genetic background of the experimental strain. At simulation time zero, exogenous *CLN2* is zero and the expression of endogenous *CLN2* and SBF activity is at background levels. This corresponds to methionine repression of exogenous *CLN2*. In full agreement with the experiment, a simulated transient expression of ectopic *CLN2* for 15 min starting at time zero is sufficient to activate SBF and to trigger the sustained expression of the endogenous *CLN2* gene. Thus, a simulation using Chen's original model ‘predicts’ that a short pulse of *CLN2* expression would be sufficient to irreversibly engage the Cln-switch in the described genetic background.

### 3.2. The Cln-switch contributes to the irreversibility of START-transition also in wild-type cells

In the wild-type situation, the Cln-switch is not isolated as in Charvin's experiments, but embedded into the global cell cycle control network. It is thus difficult to attribute a specific role to any one network motif, and we argue that the role of the Cln-switch in the START transition needs to be understood in the context of the global control system. In particular, Clb2 activation by Cln2 is expected to negatively influence the Cln-switch in the wild-type, but not in the mutant strain used in the experiments. Chen's model is a validated and exceptionally well-tested tool, which, owing to its comprehensiveness, allowed us to simulate the behaviour of wild-type cells, in which the Cln-switch is embedded within the global cell cycle control system. In a wild-type background, Clb2 activity is inhibited by Sic1 early in G1. Late in G1, high Cln2 activity releases this inhibition, and Clb2 auto-activation causes the Clb-switch to fire. Figure 2*b* shows a time course simulation of a cycling wild-type cell. In contrast to the mutant strain used in Charvin's experiments, the wild-type shows typical oscillations of Cln2 and Clb2 as a function of time. The temporal order of cell cycle progression arises from Cln2-inducing Clb2 activity, and Clb2-repressing Cln2 activity. At later stages in the cell cycle, Clb2 activity decreases mainly owing to Clb1/2 degradation by APC [26], which is initiated by Cdc20 and sustained by Cdh1. This eventually resets the system to low cyclin/Cdk1 activity.

To obtain an understanding of the relationship between the Cln-switch and the Clb-switch, we performed bifurcation analyses. To illustrate the results, we overlay two one-parameter bifurcation diagrams to obtain a pseudo-phaseplane: Because the system has many more than two state variables, a phase-plane analysis is not possible. We can, however, imagine what a phaseplane would look like, if we fix all but two state variables to their respective steady-state values. To do this, we first plot a bifurcation diagram using Clb2 as the only state variable, with Cln2 as a bifurcation parameter (figure 2*c*, black curve). All other state variables in the control system are at steady state (i.e. their differential equations are set to zero). Similarly, we can plot a one-parameter bifurcation diagram using Cln2 as a state variable and Clb2 as a bifurcation parameter (figure 2*c*, red curve). In this way, we obtain two pseudo-nullclines. Although the dynamics of the system are only approximated by the pseudo-nullclines, the steady states at the intersections of the Cln2 and Clb2 pseudo-nullclines represent the true steady states of the control system (figure 2*c*, green circles).

Because both Clb2 and Cln2 are bistable variables, both curves are S-shaped and have two stable branches connected by one unstable branch. The system has three steady states: one stable and two unstable (figure 2*c*, solid and faint green circles, respectively). A cycling cell follows the blue trajectory. The cell cycle starts close to the origin, where all cyclins are low (early G1 phase). If the cell has become big enough, Cln3 and Bck2 start to activate Cln2, and the system moves to the right. Once the Cln-switch engages and Cln2 activates itself through SBF, the trajectory shoots over a threshold determined by the Clb2 bifurcation curve, towards very high Cln2 activity. This, however, induces Clb2 activity through Cln2-dependent inhibition of Sic1 and Cdh1, which allows Clb2 to increase, causing the trajectory to turn upwards, and, because Clb2 inhibits SBF (and thus Cln2), also to the left. The trajectory is attracted by the only stable steady state, which is found at high Clb2 and low Cln2 activity (Clb-switch ON, Cln-switch OFF). In wild-type cells, this stable steady state is, however, never reached, because later events in the cell cycle, such as degradation of Clbs by the APC, cause the trajectory to bend down (not shown) and to eventually return to its origin. The cell cycle is completed.

In the wild-type situation, the Cln2 bifurcation curve crosses the abscissa only once, namely at high Cln2 (figure 2*d*, red curve; same as figure 2*c*, red curve). Thus, in the absence of any Clb2 activity, as would be the case in Sic1-4A cells, Cln2 is high and mono-stable. In Charvin's experiment, which uses Sic1-4A cells, the Cln2 switch is kept bistable only by the additional lack of Cln3 and Bck2: in *cln3*Δ*bck2*Δ**, the Cln2 bifurcation curve is shifted downwards, because Cln3 and Bck2 cannot support the ON state of the Cln-switch. The saddle nodes of the Cln2 nullcline are thus shifted to lower values of Clb2 and Cln2 can be bistable even in the absence of Clb2 (figure 2*d*, green curve). Thus, Charvin's experimental evidence suggests that, if experimentally isolated from other events, the Cln-switch is the predominant contributor to the irreversible START transition. Our theoretical analysis of START, performed in the context of a full cell cycle control system rather than an isolated switch, confirms the importance of the Cln-switch for the START transition, but additionally highlights how the function of the Cln-switch depends on the state of the Clb-switch. Our findings suggest that a robust START transition is an emergent property of several interacting network motifs rather than the consequence of any one such motif.

### 3.3. Commitment is initiated by the interaction of two bistable switches

**Figure 3.**
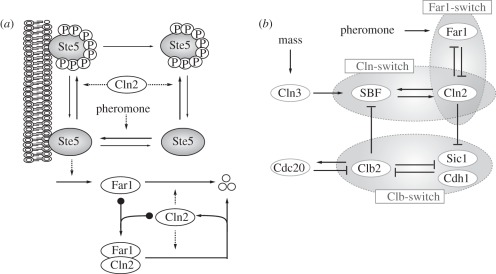
Pheromone signalling and its interaction with the cell cycle engine. Cln3, Cln2 and Clb2 denote the corresponding Cyclin:Cdk1 complexes. (*a*) Pheromone signalling. The scaffolding protein Ste5 is recruited to the plasma membrane in response to pheromone and activated. Only Ste5 that is not phosphorylated by Cln2, however, is able to act as a scaffold to promote the activation of Far1. Cln2 phosphorylates Far1 to promote its degradation, while Far1 inhibits Cln2 activity. (*b*) The Far1-switch. Mutual antagonism between Far1 and Cln2 is predicted to generate an additional bistable switch (Far1-switch). The Cln-, Clb- and Far1-switches and their interactions are shown.

Having analysed the START transition within the comprehensive framework of Chen's model, we focused our attention on a related set of problems, namely how cell cycle commitment (i.e. pheromone resistance) is initiated and maintained once START has been passed. The main link between pheromone signalling and the cell cycle engine is Far1 [7], which interacts with the cell cycle controller Cln1/2:Cdk1 in a double-negative feedback loop. Far1 inhibits Cln1/2/3:Cdk1 complexes [21,27], while Cln1/2:Cdk1 complexes inhibit Far1 by enhancing its degradation [7,12], and by preventing both Far1 synthesis and activation (figure 3*a*) through phosphorylation and inhibition of the upstream scaffolding protein Ste5 [11].

On the basis of the well-studied mutual antagonism between Cln2 and Far1, we propose the existence of an additional bistable switch, which we refer to as the Far1-switch. The differential response of pre-START and post-START cells to pheromone suggests that Far1 interacts differentially with the cell cycle engine at different stages of the cell cycle. We aimed at explaining these phenomena in terms of the Cln-switch, the Clb-switch and the proposed Far1-switch (figure 3*b*).

**Figure 4.**
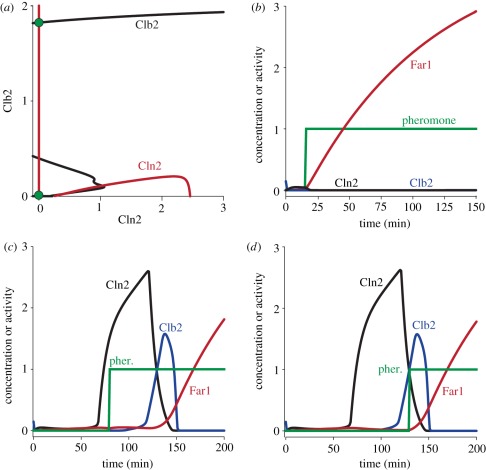
The effect of pheromone on cell cycle progression. (*a*) Bifurcation analysis. As in figure 2*c*, but including the pheromone response. Pheromone signalling pushes the Cln2 bifurcation curve down to lower Clb2 activity, which generates a new steady state not observed in the absence of pheromone signalling (compare figure 2*c*). This steady state corresponds to the pheromone-arrested state early in G1. (*b*–*d*) Time course simulations. (*b*) Pheromone is added before the START transition (early in G1), when both Cln2 and Clb2 activities are low. The cell is sensitive to pheromone and arrests early in G1. (*c*) Pheromone is added shortly after the START transition (late in G1). The Cln-switch has already fired, and Cln2 activity is high enough to prevent Far1 activation. The cell is resistant to pheromone, Far1 activation is prevented. After the Clb-switch has engaged and Cln2 activity has been sufficiently reduced, Far1 can accumulate. (*d*) Pheromone is added at a time when Cln2 activity is already fairly low; the cell cycle is not yet completed, however. In this phase, Far1 gets activated immediately after pheromone addition. This, however, does not impair completion of the cell cycle, which is now driven by Clb2 activity and no longer requires Cln2 activity.

After incorporation of Far1 into Chen's model (for details, see §5.6 and table 1), we first tested how the pseudo-phaseplane shown in figure 2*c* would change in the presence of pheromone (figure 4*a*). Because Far1 inhibits Cln2, the Cln-switch is impaired and it is easier for Clb2 to turn it OFF. The saddle nodes of the Cln2 bifurcation curve are thus shifted to lower Clb2 values by pheromone, whereas the Clb2 bifurcation curve is unaffected. As a consequence, a stable steady state emerges at low Cln2/Clb2 levels (compare figure 4*a* with figure 2*c*). In cells with low Cln2 levels, the system will be attracted by this steady state, which corresponds to the experimentally observed G1 arrest of pre-START cells with low Cln2 and low Clb2. A cell with sufficiently high Cln2 activity, by contrast (i.e. a post-START cell), will be initially attracted by the stable branch of the Cln2 bifurcation curve found at high Cln2 levels, following a trajectory similar to the one shown in figure 2*c*. Despite the presence of pheromone, such a cell is committed to finish the on-going cycle to eventually settle in the only steady state—it will arrest in G1 of the following cycle.

**Table 1.** Model equations for Far1 activation by pheromone and its interaction with the cell cycle engine. Subscripts ‘T’ and ‘M’ indicate ‘total amounts’ and ‘membrane-bound’, respectively. All rate constants k are given in min^−1^. See figure 3*a* for a cartoon representation of the interactions.

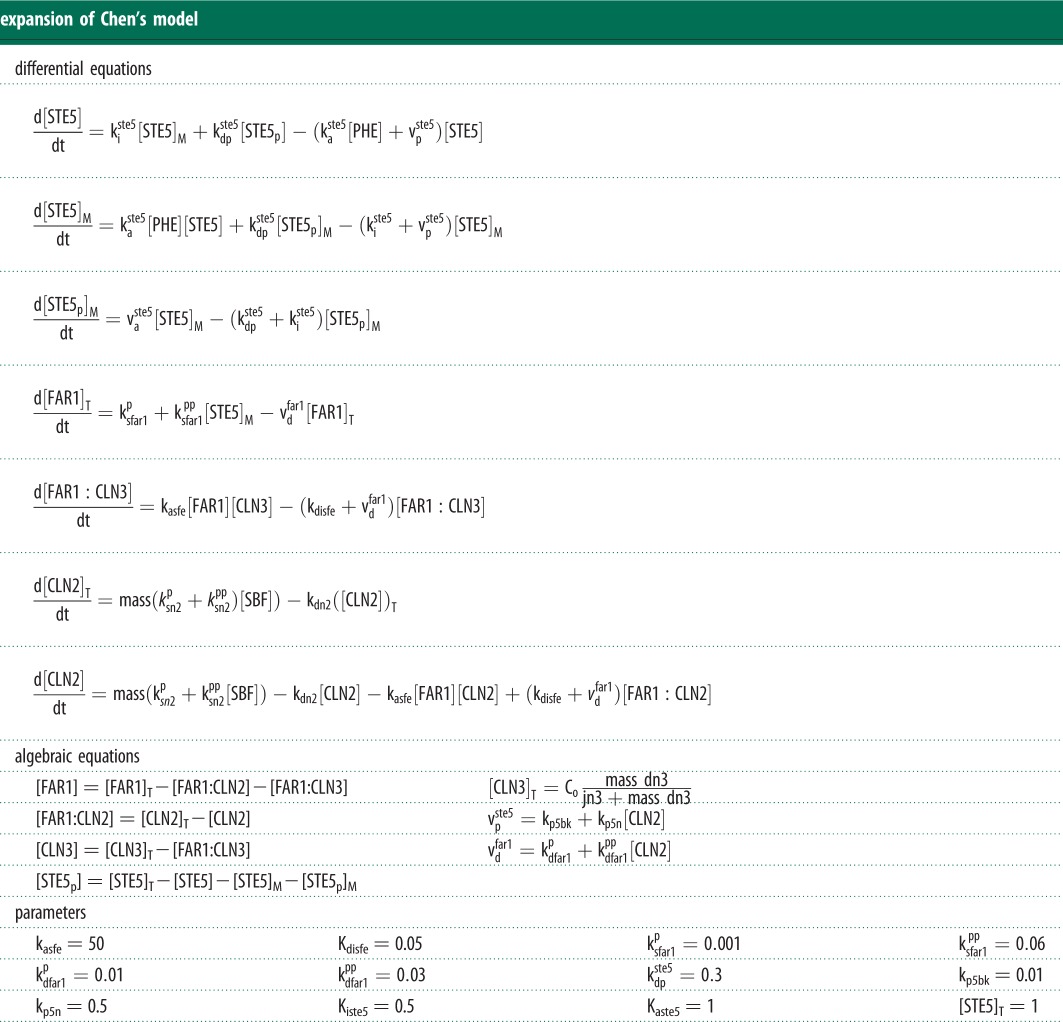

In summary, our dynamical systems analysis of the interaction of the Cln-switch and the Far1-switch visualizes how pheromone blocks the START transition, and why cells are resistant when Cln2 activity is high. One apparent problem remains, however—at later stages of the cell cycle, Cln2 is repressed by negative feedback from the engaging Clb-switch, which should restore Far1 activity. How is commitment to cell division maintained at this later stage?

### 3.4. Distinct modes of resistance to pheromone sustain commitment

In the framework of Chen's model, the answer is simple: Cln2 causes the Clb-switch to fire. Because Clb2 self-activates, this process is irreversible; Cln2 activity is no longer required for Clb2 activity. Thus, cell cycle progression becomes independent of Cln2 and is driven by Clbs only. The Clb self-activation seems strong enough to maintain cell cycle progression even in the presence of pheromone signalling [28]. Time course simulations using Chen's modified model reveal a three-stage response to pheromone, depending on the cell cycle stage. If pheromone is added prior to the START transition when Cln2 is still low, Far1 becomes activated immediately upon pheromone addition and prevents the Cln-switch from firing. The cell stably arrests in G1 (figure 4*b*). If pheromone is added after START, however, our simulations reveal two time windows with qualitatively different responses, depending on the post-START cell cycle stage: In the first phase, when Cln2 activity is still high (figure 4*c*), addition of pheromone has no immediate effect on Far1, because Cln2 efficiently blocks Far1 activation. The cell is resistant to pheromone and remains committed to cell division. In the second, later phase, however, the Clb-switch has engaged and has inhibited Cln2 activity, which is now low. Pheromone addition at this late stage thus activates Far1 immediately (figure 4*d*), similar to the situation in pre-START cells, which also have low Cln2 activity. However, because cell cycle progression at this stage is no longer driven by Cln2, but by Clb2, which is not inhibited by Far1, the cell proceeds and finishes its cycle to finally arrest in the G1 phase of the next cycle.

This second mechanism of pheromone resistance has potential biological implications, especially for large mother cells. After cell division, the mother cell experiences only a very short G1 phase, because it is nearly big enough to start another cycle. We propose that firing of the Far1-switch already prior to the completion of cell division gives Far1 a head start over Cln2 activity, and thus ensures robust arrest in G1 in the presence of pheromone. Without this head start, the outcome of the mutual antagonism between Far1 and Cln2 activity would be uncertain, potentially jeopardizing robust G1 pheromone arrest.

### 3.5. Far1-S87P and Ste5-8A compromise the Far1-switch in distinct ways

**Figure 5.**
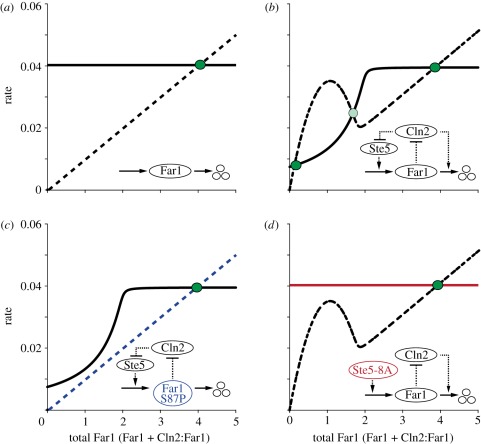
Rate plots suggest that the bistability of the Far1-switch is less robust in two mutants. All simulations were done at constant Cln2 levels and in the presence of pheromone. For parameter values, see table 1. The Far1 production rate (solid lines) and the Far1 degradation rate (dashed lines) are plotted as functions of total Far1 concentration. The network motifs corresponding to the plots are shown in insets. (*a*) No feedback regulation. In the hypothetical case of unregulated Far1 production and degradation, the Far1 production rate is a horizontal line (constant, zero-order synthesis). The degradation rate is proportional to the total concentration of Far1 and its slope represents the degradation rate constant (first-order degradation kinetics). (*b*) Wild-type with two double-negative feedbacks. Cln2 exerts dual control by repressing Far1 activation and promoting Far1 degradation, creating two double-negative feedback loops. Because of the shapes of the curves, production and degradation rates are highly likely to intersect three times for a wide range of parameters to create two stable (solid green circles) and one unstable (faint green circle) steady states. This suggests robust bistability in the wild-type case. (*c*) Far1-S87P with one double-negative feedback loop. The Far1-S87P degradation rate is not enhanced by Cln2 and is thus directly proportional to the total amount of Far1-S87P (blue-dashed line). The Far1-S87P production rate is the same as for the wild-type. The shapes of the curves in principle still allow three intersections, albeit only in a restricted range of parameters. For most parameter sets, the bistability is lost, as is exemplified here. This finding suggests less robust bistability in the Far1-S87P mutant when compared with wild-type. (*d*) Ste5-8A with one double-negative feedback loop. In Ste5-8A cells, Far1 production rate is no longer repressed by Cln2 and is constant. The Far1 degradation rate is as in the wild-type. Similar to Far1-S87P, bistability is still possible, but predicted to be less robust.

To address the behaviour of mutant cells, in which Far1 inhibition by Cln2 is compromised, we analysed the Far1 network motif in greater detail. Interestingly, Cln2 inhibits Far1 through two arms, preventing Far1 production on the one hand, and promoting Far1 degradation on the other (figure 5). Two mutants have been described that disrupt either one or the other arm of Cln2-dependent Far1 inhibition: non-phosphorylatable Far1-S87P, also known as Far1-22p, which is resistant to Cln2-induced degradation [12]; and Ste5-8A, which lacks eight Cln1/2:Cdk1 phosphorylation sites and, in the presence of pheromone, is constitutively active [11].

To understand the effects of these mutants on the Far1-switch and on commitment, we analysed the Far1/Cln2 module in isolation (for the equations, see table 1). An illustrative way of looking at the effect of these mutants is rate plots (figure 5). Production and degradation rates of total Far1 are plotted as functions of total Far1 concentration. Steady states are found at the intersections of the two curves. In the hypothetical case of unregulated Far1 synthesis and degradation, the synthesis rate is constant and independent of the total Far1 levels, whereas the degradation rate is a line for which the slope corresponds to the first-order degradation rate constant (figure 5*a*).

In wild-type cells, however, both production and degradation rates are dependent on Cln2 activity, which itself depends on Far1 levels. Thus, the production and the degradation rates change in a nonlinear manner with increasing concentrations of Far1 (figure 5*b*). If total Far1 is low, the Far1 production rate is small (figure 5*b*, solid line), because Cln2 is active, and phosphorylates and inactivates Ste5, which is required for pheromone-induced Far1 production. However, if total Far1 exceeds the level of total Clns, Clns activity approaches zero, and most Ste5 is in the unphosphorylated, active form. The Far1 production rate becomes maximal. The Far1 degradation rate also shows a nonlinear response with respect to total Far1 (figure 5*b*, dashed line): if total Far1 is smaller than Cln2, Cln2 is able to phosphorylate Far1 and accelerate Far1 degradation. If total Far1 exceeds total Cln2, Cln2 activity is negligible and Far1 is degraded close to the basal first-order rate, directly proportionally to its concentration. The curves for Far1 production rate and degradation rate intersect at three points, creating two stable (solid green circles) and one unstable (faint green circle) steady states. Thus, Far1 is bistable and, depending on the state of the system, either high or low. The rate plot suggests robust bistability in the wild-type case: the curves can change their positions substantially without losing any of the steady states. In the mutant cases, however, either one or the other rate curve becomes linear: the production rate in the Ste5-8A mutant (figure 5*c*), or the degradation rate in Far1-S87P cells (figure 5*d*). Three intersections of the curves (and thus bistability) are still possible in both mutants; however, the regions in parameter space that yield bistability are expected to be more restricted than in the wild-type. From this simple graphical analysis, we hypothesize that the seemingly redundant regulation of Far1 by Cln2 (suppressing Far1 synthesis and enhancing Far1 degradation) contributes to robust bistability and that disruption of either arm of this regulation will compromise this robustness.

**Figure 6.**
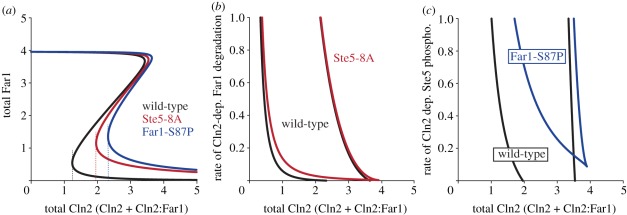
The commitment point is predicted to be shifted more markedly in Far1-S87P than Ste5-8A cells. (*a*) One-parameter bifurcation diagram. Comparison of the bistability regimes of the Far1-switch in the wild-type case (black curve) with Ste5-8A (red curve) and Far1-S87P (blue curve). In the wild-type, the bistable regime extends over a wide range of Cln2 levels. The commitment point (defined as the Cln2 activity at which the cell becomes resistant to pheromone; dashed vertical lines) is low. In the case of the mutants, the bistable regime is significantly reduced, and the commitment points are shifted to higher Cln2, more so in the case of Far1-S87P than in the case of Ste5-8A. (*b,c*) The difference in commitment point shift between Far1-S87P and Ste5-8A is a robust system property. (*b*) Two-parameter bifurcation diagram for Ste5-8A. The control parameters are the total amounts of Cln2, and the rate constant of the Cln2-dependent Far1 degradation, *k*_*dfar1,pp*_. High values of *k*_*dfar1,pp*_ indicate that Cln2-dependent Far1 degradation is fast. In this case, Ste5-8A and wild-type are very similar, because sufficient mutual inhibition between Cln2 and Far1 arises by Cln2-dependent Far1 degradation alone. At low values of *k*_*dfar1,pp*_, however, higher levels of Clns are required to enter the bistable regime in the Ste5-8A mutant than in the wild-type. (*c*) Two-parameter bifurcation diagram for Far1-S87P. Here, the second control parameter is the rate constant of the Cln2-dependent Ste5 phosphorylation, *k*_p5n_. Comparing the wild-type case with the Far1-S87P mutation yields a similar picture as in (*b*); however, the reduction of the bistable regime by the Far1-S87P mutation is more pronounced, suggesting higher importance of this arm for bistability.

To corroborate our hypothesis, we first performed a one-parameter bifurcation analysis (figure 6*a*). Indeed, the bistable regime is largest for wild-type cells, while both mutants decrease the bistable regime. If we define as ‘commitment point’ the Cln2 activity at which the cell becomes resistant to pheromone, the commitment points of both mutants are predicted to be shifted to higher Cln2 activity compared with the wild-type. Moreover, our results suggest that this effect is more pronounced in Far1-S87P cells than in Ste5-8A cells: Far1-S87P cells are expected to require higher Cln2 activity to acquire pheromone resistance and to commit to division than Ste5-8A cells. Two-parameter bifurcation diagrams (figure 6*b*,*c*) reveal that these effects are robust in terms of parameter values and are thus an emergent property of the specific wiring of the network. Importantly, this finding is consistent with recently published data, which defined the commitment point in terms of the degree of Whi5 nuclear export [14]. In qualitative agreement with our generic results, these authors found a shift of the commitment point in Far1-S87P cells, but not in Ste5-8A cells, a situation that happens in our model when Cln2-dependent Far1 degradation is fast (figure 6*b*,*c*). Thus, the experimentally observed behaviour is a particular realization of our more general description, and is entirely consistent with our model.

**Figure 7.**
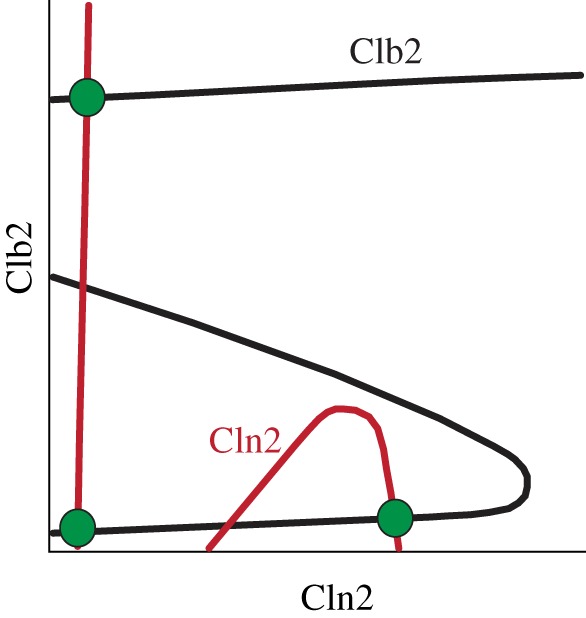
Model prediction. Hand-drawn sketch illustrating our proposed explanation for aberrant cell cycle arrest in Ste5-8A. This qualitative sketch shows how we would expect figure 4*a* to change if constitutively active Ste5 inhibited Clb2 activity independently of Far1. In such a scenario, a third stable steady state would be generated at high Cln2 activity and low Clb2 activity, corresponding to an aberrant cell cycle arrest after genome duplication, which is observed in a proportion of Ste5-8A cells treated with pheromone.

Unexpectedly, in response to pheromone, a significant proportion of Ste5-8A cells arrest post-START with 2N sets of chromosomes in a Far1-independent manner [11]. The arrest at this second block point is reminiscent of the pheromone arrest of cells deficient in Clns and overexpressing Clb5 [29]. We propose that the puzzling phenotype of Ste5-8A cells is caused by pheromone-dependent reduction of Clb2 activity. In terms of a dynamical systems analysis, compromised Clb2 activity by Ste5-8A is predicted to change the picture shown in figure 4*a* as sketched in figure 7. Clb2 inhibition in response to pheromone makes firing of the Clb-switch more difficult, and its OFF state would extend to higher Cln-levels. The Cln-switch, in turn, now lacking the negative feedback from the Clb-switch, would be more likely to reside in the ON state. In this way, an additional stable steady state is created at high Cln2 and low Clb2 activity, which might explain the arrest after genome duplication observed in a sub-population of Ste5-8A cells in response to pheromone. Importantly, the original stable steady state that corresponds to a G1 arrest is still present in these cells, which would explain why some cells arrest in G1 and some cells arrest in later stages of the cell cycle. In summary, we make the testable prediction that the proportion of Ste5-8A cells that shows an abnormal arrest in response to pheromone has high Cln2 activity, but low Clb2 activity.

## 4. Discussion

Mathematical models of biological systems often have a limited scope, because the datasets required to sufficiently constrain larger models are not available at the time of model conception. Despite its potential to overcome this problem, the frequently cited ‘virtuous cycle’ between experimentation and modelling usually ceases once a model is published, and only a few models seem to benefit from being tested against new experimental datasets as new information becomes available. This is surprising, given the broad consensus that models should be continuously challenged with novel datasets and updated accordingly in order to grow both in scope and in accuracy. Here, we use Chen's model [19] to showcase that a published model can indeed be used to analyse novel experimental findings and to gain insight into problems that were not originally considered during model conception. As an additional benefit of this approach, the mathematical model itself is increased in scope, applicability and accuracy. Only by continuous development can models remain up-to-date tools to further our understanding of biological systems.

Charvin *et al*. [13] have shown experimentally that transient expression of exogenous Cln2 triggers sustained expression of endogenous Cln2. When queried accordingly, Chen's model readily predicts this result, which was unknown at the time the model was created. This is despite the fact that Chen's model does not explicitly account for Whi5, but models the Cln-switch as a direct positive feedback between Cln2 and SBF, illustrating that, from a network perspective, this approximation can substitute for the exact molecular details. A minor expansion of the original model (Far1 ⊣ Cln2 ⊣ Far1) provided significant insight into a related (yet distinct) problem, namely pheromone resistance and cell cycle commitment. A yeast cell responds to pheromone with arrest if (i) the pheromone signal is generated and transmitted and (ii) the cell cycle engine is susceptible to the effects of such signalling. Both conditions together are required, and also sufficient, for arrest in response to pheromone. Thus, cell cycle commitment (i.e. resistance to pheromone signalling in cell cycle phases other than G1) indicates either that pheromone signals are no longer transmitted, or that the driver of the cell cycle engine that is targeted by pheromone signalling has become dispensable for cell cycle progression. Our results strongly suggest that both scenarios are realized and contribute to cell cycle commitment, albeit at different stages of the cell cycle: shortly after the START transition, the cell is resistant to pheromone because pheromone signalling is actively repressed by the cell cycle engine, Cln2 inhibiting Far1 synthesis and facilitating its degradation. At later stages, Cln2 activity is no longer sufficient to repress the Far1-switch. Pheromone signalling is active at this stage, but the cell remains committed to division, because the target of pheromone signalling, Cln2 activity, is now no longer required for cell cycle progression, which at this stage is driven by Clb2.

Our model describes the interaction of Far1 and Cln2 by redundant double-negative feedbacks via stoichiometric complex formation. The model predicts a novel bistable Far1-switch, which cooperates with the Cln-switch in order to create a robust decision-making process at START. Introduction of this hypothetical switch allows our model to preserve a bistable response, even if the Cln-switch is compromised, as is the case, for instance, in *whi5*Δ** mutants. The uniform G1 arrest of *whi5*Δ** mutants at high pheromone concentration [30] provides strong evidence for the postulated bistable Far1-switch of our model, which is not present in previous models [14].

In summary, the incorporation of the mutual antagonism between Far1 and Cln2 into Chen's model strongly suggests a contribution of Clb2 in conferring pheromone resistance (and thus cell cycle commitment) at late stages of the cell cycle. Our analysis of a comprehensive cell cycle model provides evidence that cell cycle progression and robust commitment in early and late post-START cells are controlled by multiple interconnected bistable switches, both in the cell cycle control network itself, and in the way pheromone signalling interacts with this network. As an extended value, we hope this work will encourage the application, adaptation and expansion of published models, to keep these models alive and up to date. We believe this would greatly accelerate the progress towards the ultimate aim of developing comprehensive, realistic descriptions of functional modules of the cellular protein interaction network.

## 5. Methods

### 5.1. Ordinary differential equation-based modelling

In our approach, the dynamics of a molecular regulatory network is described by a system of ordinary differential equations (ODEs). ODEs describe the rate of change of components that characterize the state of the biochemical system (state variables). The right-hand side of each ODE contains positive (synthesis and activation) and negative (degradation and inactivation) terms for each of the reactions in which a biochemical species participates as a product or a reactant. To calculate how fast each component changes with time, the system of ODEs is integrated numerically. Such integration requires prior knowledge of the numerical values of every single kinetic parameter in the model.

### 5.2. Dynamical systems theory: rate plots and bifurcation diagrams

Because these parameter values are rarely measured by direct experimentation, we use dynamical systems theory (DST) as a method to qualitatively determine the dynamic properties of a molecular regulatory system before we know (or estimate) the values of the kinetic parameter. Essentially, DST is a toolkit for dynamic network analysis. For simple networks, a useful first step is to plot the rates of production and removal of a particular component, X, as functions of its concentration, [X]. Wherever the two curves intersect, [X] assumes a steady-state value (figure 5). For networks with feedback loops, there can be more than one intersection, corresponding to stable and unstable steady states, as is the case, for instance, in figure 5*b*. By computing these steady-state values as functions of a ‘signalling’ parameter, we plot a signal–response curve. In the DST terminology, the signal–response curve is called a one-parameter bifurcation diagram, and the ‘signal’ is called bifurcation parameter or control parameter.

### 5.3. Dynamical systems theory: phase-plane analysis

A signal–response curve can also be thought of as a balance curve for a dynamic variable, indicating at which signal levels the response variable is in steady state, because its production is exactly balanced by its consumption. In cases where the response variable feeds back on the signalling system, it is natural to reverse the roles of ‘signal’ and ‘response’. Plotting the two bifurcation curves on the same coordinate systems generates a phase plane. The two curves on the phase plane are called nullclines, because along these curves one of the dynamical variables is not changing in time (its time-derivative is zero). For multidimensional systems, a phase plane can be calculated by assuming that all, except two, dynamic variables are in (pseudo) steady state. This is a useful way to project a multidimensional control system onto a two-dimensional plane. Pseudo-phase planes can be useful in interpreting the origins of multiple steady states and limit cycle oscillations.

### 5.4. Chen's model

Chen's model [19] can be downloaded from the model website at http://mpf.biol.vt.edu/research/budding_yeast_model/pp/ or simulated online. The webpage also contains detailed information on the simulations for wild-type cells and a wide range of mutants. All simulations were performed using the originally published parameter set [19], which successfully captures the qualitative behaviour of more than 130 mutant strains.

### 5.5. Simulation software

Simulations and bifurcation analyses were performed with the open access software packages Oscill8 (http://oscill8.sourceforge.net/) and XPPAUT (http://www.math.pitt.edu/~bard/xpp/xpp.html). Discrete events were removed from Chen's model in all bifurcation computation. Cell mass was set to mass = 2, corresponding to a big mother cell.

### 5.6. Computation of the figures

To simulate Charvin's experiment (figure 2*a*), Chen's model was modified to match the experimental strain and conditions. Expression of *GAL1-SIC1-4A* was modelled by setting *k′*_sc1_ *=* 0.12, MDT = 150, *V*_kp,c1_ = 0 and *k*_pp,c1_ = 0. The *Δcln3*Δ*bck2* background was accounted for (*Bck2 =* 0 and *Cln3 =* 0). For ectopic Cln2, we added an additional equation with a binary production rate and a degradation rate matching the rate of endogenous Cln2. Figure 2*b* is simulated using the original Chen model and parameters. In figure 2*c*, the black Clb-bifurcation curve was calculated at constant Cdc20 to remove the Clb2-Cdc20 negative feedback loop (*cdc20 =* 0.3). This is required to reveal the bistable switch. The red Cln-bifurcation curves in figure 2*c*,*d* are identical and were calculated using the equations for Cln2, SBF, Bck2 and Cln3. The black curve in figure 2*d* is computed with *Bck2 =* 0 and *Cln3 =* 0 (figure 4). Figure 4*a* was computed as for figure 2*c*, but including pheromone signalling in the calculation of the Cln2 bifurcation curve. The additional equations are shown in table 1. Figure 4*b*–*d* were all computed with the comprehensive model (i.e. Chen's model expanded to include the Far1-Cln2 mutual antagonism). Figures 5 and 6 were computed with the pheromone signalling pathway isolated from the comprehensive model (i.e. using the equations and parameters given in table 1). Pheromone was present in the simulation. To focus on the interplay between Cln2 and Far1, Cln3 was removed (*dn3 =* 0), and Cln2 levels were set constant (*[CLN2]*_T_ = 1.8). Ste5-8A and Far1-S87P were mimicked by zeroing their phosphorylation rates (*k*_p5n_ *=* 0 and *k*_*dfar1pp*_ *=* 0, respectively). The two-parameter bifurcation diagrams were computed by tracing the saddle-node bifurcations shown in the one-parameter bifurcation diagram.
